# A Robust Multilevel DWT Densely Network for Cardiovascular Disease Classification

**DOI:** 10.3390/s20174777

**Published:** 2020-08-24

**Authors:** Gong Zhang, Yujuan Si, Weiyi Yang, Di Wang

**Affiliations:** 1 College of Communication Engineering, Jilin University, Changchun 130012, China; zhanggong18@mails.jlu.edu.cn (G.Z.); yangwy19@mails.jlu.edu.cn (W.Y.); 2 School of Electronic and Information Engineering (SEIE), Zhuhai College of Jilin University, Zhuhai 519041, China; 3 School of Electronics & Information Engineering, Tianjin Polytechnic University, Tianjin 300387, China; wangdi17@mails.jlu.edu.cn

**Keywords:** electrocardiogram (ECG), cardiovascular disease, inter-patient paradigm, robustness to noise, imbalance category

## Abstract

Cardiovascular disease is the leading cause of death worldwide. Immediate and accurate diagnoses of cardiovascular disease are essential for saving lives. Although most of the previously reported works have tried to classify heartbeats accurately based on the intra-patient paradigm, they suffer from category imbalance issues since abnormal heartbeats appear much less regularly than normal heartbeats. Furthermore, most existing methods rely on data preprocessing steps, such as noise removal and R-peak location. In this study, we present a robust classification system using a multilevel discrete wavelet transform densely network (MDD-Net) for the accurate detection of normal, coronary artery disease (CAD), myocardial infarction (MI) and congestive heart failure (CHF). First, the raw ECG signals from different databases are divided into same-size segments using an original adaptive sample frequency segmentation algorithm (ASFS). Then, the fusion features are extracted from the MDD-Net to achieve great classification performance. We evaluated the proposed method considering the intra-patient and inter-patient paradigms. The average accuracy, positive predictive value, sensitivity and specificity were 99.74%, 99.09%, 98.67% and 99.83%, respectively, under the intra-patient paradigm, and 96.92%, 92.17%, 89.18% and 97.77%, respectively, under the inter-patient paradigm. Moreover, the experimental results demonstrate that our model is robust to noise and class imbalance issues.

## 1. Introduction

Cardiovascular disease is a major health problem worldwide. According to recent data from the World Health Organization, 30% of the 58 million deaths worldwide are due to cardiovascular disease [1]. Fortunately, early diagnosis and symptomatic treatment of cardiovascular disease can reduce mortality by more than 70%. Therefore, early accurate diagnosis of cardiovascular disease is critical to saving patients’ lives.

Coronary artery disease (CAD) is one of the most typical cardiovascular diseases. It is mainly the result of atherosclerosis, in which fibrous plaque begins to form a thick area on the inner wall of the artery, leading to slowing down the flow of blood to the heart [2,3]. In severe conditions, CAD can lead to myocardial infarction (MI) or congestive heart failure (CHF), if it is not diagnosed in time. Electrocardiogram (ECG) is the most commonly used diagnostic tool because of its non-invasiveness and low cost. Usually, doctors evaluate ECG signal morphology and its characteristics in order to make clinical decisions on CAD, MI and CHF [4].

The ECG performance of patients with CAD will mainly have abnormal T-wave conditions. In general, myocardial ischemia manifests as ST elevation, on the contrary, subendocardial ischemia is reflected in ST-segment depression [5]. For left main coronary artery occlusion (LMCA) in patients with MI (circumflex arteries and left anterior descending artery (LAD)), the most prominent ECG waveforms are depressed ST segments in leads I, II, V4, V5, and V6, while ST segments in lead aVR are elevated [6]. The presence of occlusions in the LMCA and proximal LAD arteries is reflected by an ST elevation > 1 mm in the aVR lead. If myocardial infarction and its location are not captured in time, MI may further damage left ventricular (LV) function, resulting in LV dysfunction. It is reflected in the QRS complex number of the ECG waveform as a low amplitude, indicating the presence of CHF [7].

However, CAD can be hard to diagnose since the early stages have no visible symptoms, which means manual interpretation of these ECG morphological changes and features is very difficult. In addition, processing huge ECG signals and their overlapping characteristics is time-consuming. Therefore, computer-aided technology is required to overcome these limitations.

## 2. Related Works

In this section, we first discuss the two main evaluation paradigms of heartbeat classification, the intra-patient and inter-patient paradigms. Next, the existing ECG classification methods shown in Table 1 will be introduced. Subsequently, we propose our model and briefly explain its advantages. Finally, we illustrate the structure of this paper.

### 2.1. Evaluation Paradigms

Under the intra-patient paradigm, the heartbeat of the same patient is used to train and test the heartbeat classifier. A model under intra-patient paradigm will achieve great performance during the test phase because it is well known for producing biased results by learning the characteristics of each patient during the training phase [24]. However, the trained model must deal with the heartbeat of a patient who is invisible during training in a real scenario. In contrast, the inter-patient paradigm means that the heartbeats for training and test data sets come from different patients [25]. The same heartbeat classification methods evaluated under the intra-patient paradigm shows significantly higher accuracy than under the inter-patient paradigm [26].

### 2.2. Existing Methods

In recent years, many algorithms for automatic detection and classification of ECG heartbeat patterns have been presented in the literature. Table 1 summarizes the various detection and diagnostic studies for normal and CAD, normal and MI, normal and CHF, and all of them. In these studies, the system generally consists of three steps, which are described in pre-processing, feature extraction, and classification. The pre-processing stage usually experienced three stages from noise removal, the R-peak detection and heartbeat segment. Then, many signal processing techniques, such as wavelet transform [9,17] neural networks [10,13,18], will be used to extract desirable features from ECG heartbeats. Finally, these features are classified by various classifiers such as support vector machines [16], K-Nearest Neighbors [8,17,19,21,22] and so on. 

Although many methods were identified for the detection of cardiovascular disease, there still exists space to further enhance. First, according to the literature [21,22], we can find that the performance for automated detection of 4-class cardiovascular disease still has room to improve. Second, conventional preprocessing operations (noise removal, R-peak detection, and heartbeat segment) were adopted by most researchers, we can find a simpler way to get a desirable input form. Third, all the related studies we reviewed did not show the performance of their model in different noise environments and lacked an assessment of the robustness to noise. More importantly, few studies evaluated their methods under inter-patient paradigm although they have achieved great performance under intra-patient paradigm [9,12,20]. As we know, studying for the detection of cardiovascular disease under inter-patient paradigm is more important for practical use.

### 2.3. Proposed Method and Arrangement

In this paper, we present a novel and effective model (MDD-Net) for the detection of cardiovascular disease. Here are the reasons for choosing our methods.

First, most of the existing methods [17,18,19] depend on data preprocessing, such as noise removal and R-peak location. We work on obtaining effective input data by using a light preprocessing method and still have the same level or better performance as the latest method. In this paper, we proposed an adaptive sample frequency segmentation algorithm (ASFS). Using this method, we can obtain a unified and effective input form from databases with different sampling frequencies.

Second, many feature extraction methods can effectively extract the corresponding features and achieve high performance; for example, various wavelet transforms [17,19,23] can extract time-frequency domain features, while deep learning methods [10,14,18], can extract locations and abstract features. Is it possible to increase stability and general performance by combining multiple types of features? We have made many attempts to answer this question and found that by using the concept of multilayer dense connections, the combination of abstract features from DenseNet and time-frequency domain features from multilevel DWT can achieve excellent performance in intra-patient scenarios and stable generalization performance under inter-patient conditions.

Third, many existing methods have attempted to address the category imbalance in models by adding synthetic data or adjusting the sample weights. In this paper, we use a hybrid method based on the Borderline-SMOTE algorithm to increase the training set size and reduce the internal weights of simple samples with a focal loss function. The experimental results show the good effectiveness and accuracy of the hybrid method.

The remainder of this paper is organized as follows. Section 3 first introduces the database used in this paper and gives the structure of the proposed system. Then, we show the input data generation in detail and explains the basic theory of the proposed framework. The experimental results and discussion are present in Section 4 and Section 5, respectively. The last chapter summarizes the paper and illustrates the study’s results and real significance.

## 3. Materials and Methodology

### 3.1. Data Used

In this work, we used three open access databases, PTB Diagnostic ECG Database [27] (ptbdb), St Petersburg INCART 12-lead Arrhythmia Database [27] (incarddb), and BIDMC Congestive Heart Failure Database [27] (chfdb), which were downloaded from PhysioBank [27]. We collected a total of 52 normal subjects, 148 MI subjects from the ptbdb, 7 CAD subjects from incarddb, and 15 subjects from chfdb. Only lead II in each database was used as experimental data. Table 2 summarizes the details about the data used in this paper.

For a fair comparison, we apply 10-fold cross-validation for the intra-patient paradigm according to [23]. Since we did not find literature to describe the data distribution for the 4-class cardiovascular disease under the inter-patient paradigm, we split all records into training and testing sets just similar to the method in [24], in which the subjects of training and testing are nearly in the same proportions. Details of the data distribution scheme are summarized in Table 3.

### 3.2. The Proposed System

**Figure 1:**
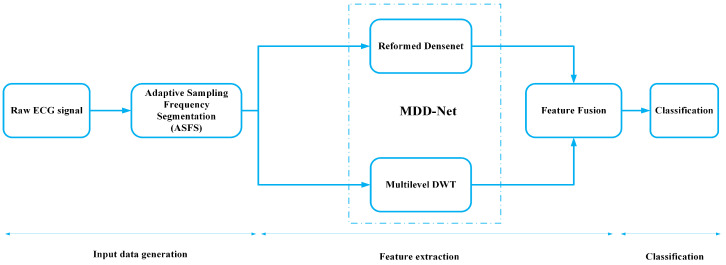
Block diagram of the proposed system.

The block diagram of the proposed system is shown in Figure 1. The system consists of three phases, which are described in input data generation, feature extraction, and classification. The working of each block is explained in detail in the following sections.

### 3.3. Input Data Generation

**Figure 2:**
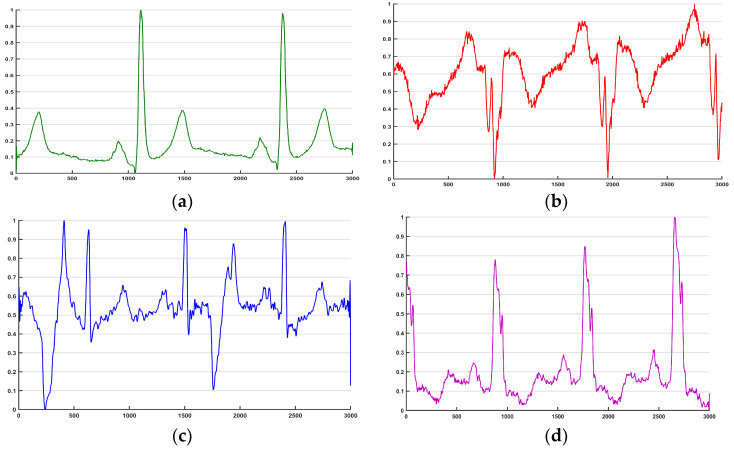
The waveform of one ECG segment. (**a**) normal; (**b**) MI; (**c**) CAD; (**d**) CHF.

We used three data sets of ECG signals with frequencies from 250 Hz to 1000 Hz. We propose an ASFS algorithm to obtain ECG segments without using regular preprocessing operations (denoising, R-peak localization, and heartbeat segmentation). The implementation flow chart is given in Table 4. From the flow chart, we extract segments containing the same number of periodic rhythms from data sets with different frequencies. Then, to ensure the quality of the signal segmentation process, segments from CAD and CHF are upsampled to the maximum sample frequency (1000 Hz) with interpolation. Note that there is a certain overlap between two segments. Using this overlap not only increases the number of training samples but also allows the convolutional network to learn features from both periodic and inter periodic perspectives. The waveforms from the different segments are shown in Figure 2.

### 3.4. Feature Extraction (Multilevel DWT)

**Figure 3:**
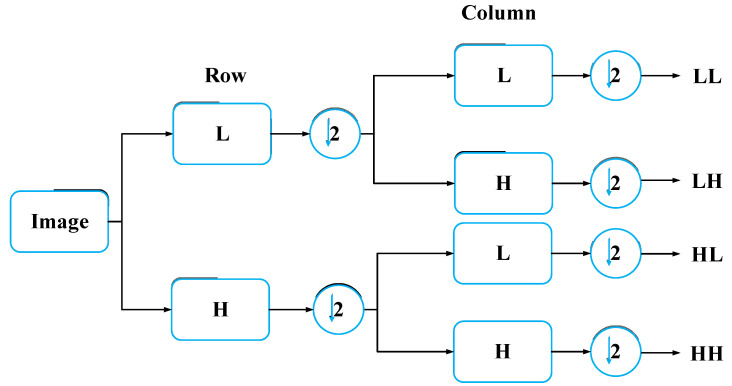
One-level wavelet transform for image.

**Figure 4:**
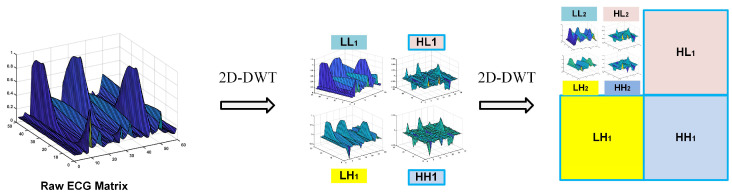
Two-level 2D-DWT for raw ECG matrix.

DWT technology converts time-domain signals into the wavelet domain to obtain both frequency and location features [28]. By using DWT, the ECG signal is divided into different scales by high-pass filtering and low-pass filtering [29]. In this paper, we fold an ECG segment into a two-dimensional matrix, which can be regarded as a single-channel gray image. For image wavelet transform, DWT should be extended to two-dimensional discrete wavelet transform (2D-DWT), which involves low-pass and high-pass filters in both the horizontal and vertical directions. This process is described in Figure 3. L and H represent one-dimensional low-pass and high-pass filters, respectively. After a one-level wavelet is transformed, the original image is transformed into four sub-images, including the approximate image (coefficients) LL and three detailed images (coefficients) HL, LH, and HH. As Figure 4 shows, we decompose the two-level one-channel image (ECG matrix) by wavelet decomposition. Note that the approximation coefficients (LL) are generally further decomposed as it represents the most useful information of the original image. Since we chose the Harr wavelet basis, the width and height of each wavelet-transformed image are halved. Profiting from the idea of the densely net, we concatenate all feature maps of decomposed images as the input of the reformed DenseNet. In the next section, we explain the structure of the proposed model.

### 3.5. Feature Extraction (MDD-Net)

**Figure 5:**
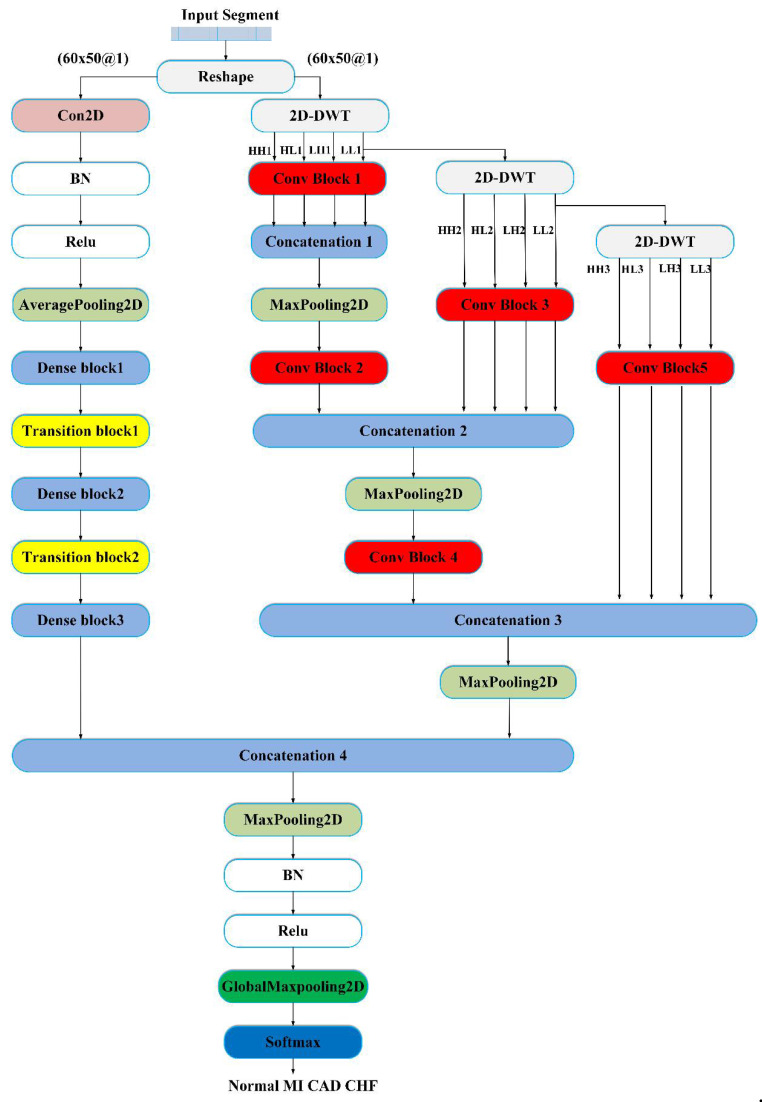
Flowchart of the proposed deep learning framework (MDD-Net) for cardiovascular disease. BN = Batch Normalization, Relu = Rectified Unit Activation.

In the field of computer vision, CNNs, such as the recent VGG-Net [30], GoogLeNet [31], Inception [32], and other models, are now commonly used. A milestone in CNN history was the emergence of the ResNet [33] model. The core of the ResNet model is establishing “shortcuts and skip connections” between the front and back layers, which is helpful in the backpropagation of the gradient in the training process used to train a deep CNN. Benefiting from the basic concept of ResNet, DenseNet [34] establishes dense connections between among the front layers and the back layers. Compared with ResNet, DenseNet has fewer parameters and mitigates vanishing gradient and model degradation issues since there are direct connections from the low- to high-level layers, which can be represented as follows:

(1) $$x_{l}=H_{l}\left(\left[x_{0},x_{1},\ldots ,x_{l-1}\right]\right)$$

where $H_{l}\left(•\right)$ represents a nonlinear transformation, which may include a series of BN, ReLU, pooling, and convolution operations. $\left[x_{0},x_{1},\ldots ,x_{l-1}\right]$ is the concatenation of feature maps from all previous layers into a single tensor, and $x_{l}$ is the output of the l th layer. Note that there may be multiple convolutional layers between layer l and layer l−1. Figure 5 shows the structure of the proposed MDD-Net, which consists of two models: the reformed DenseNet and a multilevel DWT model. The reformed DenseNet model is mainly composed of 3 dense blocks and 2 transition layers. The multilevel DWT model, including the convolution block, pooling, and concatenation modules, performs three levels of decomposition. After the last dense block, the feature maps from DenseNet and the feature maps from the multilevel DWT are concatenated. Finally, maxpooling, global maxpooling, and softmax classifiers are combined to reduce the feature dimensions and classify disease labels. The detailed network structure of MDD-Net is shown in Table 5.

### 3.6. Robustness to Imbalance Category (Borderline-SMOTE and Focal Loss Function)

Class imbalance refers to a situation in which the number of training samples in different categories used for classification tasks varies greatly. In realistic learning and classification tasks, we often encounter category imbalance. In this work, the number of normal cases is less than the number of cases for other diseases, which does not correspond to reality. Hence, we also tested the classification performance of the proposed method under different ratios of classes. To obtain good performance with imbalanced classes, we adopted the combination of the Borderline-SMOTE method and a focal loss function. The Synthetic Minority Oversampling Technique (SMOTE) [35] is an improved algorithm for random oversampling. Since random oversampling directly reuses a small number of classes, many duplicate samples are included in the training set, which may lead to overfitting. The basic idea of the SMOTE algorithm is to randomly select a sample $x_{i}$ for each minority-class sample $x_{i}^{\prime }$ and then randomly select a point on the line between $x_{i}$ and $x_{i}^{\prime }$ as the newly synthesized minority-class sample. Han et al. [36] proposed Borderline-SMOTE to solve the problems of marginalization and blindness in the SMOTE algorithm. The flow of the Borderline-SMOTE algorithm is shown in Table 6.

Furthermore, to balance the contributions of different samples to the model, we adopted the focal loss function (*FL*) proposed by Lin et al. [37]. This function was modified based on the standard cross-entropy loss (*CE*), and it can reduce the weights of easy-to-classify samples so that the model can focus on hard-to-classify samples during training. The *CE* loss function formula is as follows:

(2) $$CE(p,y)=\left\{ \begin{array}{ll} -\log\left(p\right) & if\;y=1\\ -\log\left(1-p\right) & otherwise. \end{array}\right.$$

where y is the label of the true sample (1: positive and 0: negative) and p is the category prediction probability, which ranges from 0 to 1. The larger the output probability is, the smaller the loss is for positive samples. For negative samples, the smaller the output probability is, the smaller the loss. The *CE* function in some cases may be relatively slow to iteratively run for large numbers of simple samples and may not be optimal. Hence, *FL* is presented as follows:

(3) $$FL(p,y)=\left\{ \begin{array}{ll} -\alpha {\left(1-p\right)}^{\gamma }\log\left(p\right) & if\;y=1\\ -\left(1-\alpha \right)p^{\gamma }\log\left(1-p\right) & otherwise. \end{array}\right.$$

Compared with CE, FL includes the factors γ and α. If γ>0, the loss of easy-to-classify samples will be reduced, and the model focuses on difficult to classify and misclassified samples. If γ=0, the function is simplified to the *CE* loss function. α is used to balance the uneven proportions of positive and negative samples.

In this work, the Borderline-SMOTE algorithm is used to increase the number of valid samples in the training set if the ratio of samples in disease and normal categories is less than 1/3. Moreover, the *FL* function is used when class imbalance occurs; otherwise, *CE* function is used.

### 3.7. Classification

Softmax [38] is widely used in machine learning and deep learning. The output unit of the final classifier needs softmax function for numerical processing. The softmax function [38] is defined as follows:

(4) $$S_{i}=\frac{e^{V_{i}}}{\sum_{i}^{C}e^{V_{i}}}$$

where *V_i_* is the output of the previous output unit of the classifier. *i* represents the category index, and the total number of categories is *C*. *S_i_* represents the ratio of the index of the current element to the sum of the indices of all elements. Softmax converts multi-class output values into relative probabilities for easier understanding and comparison.

### 3.8. Evaluation Index

In this paper, we use accuracy, sensitivity, specificity and overall accuracy as the main evaluation indexes, in which *TP* means detection correctly with the disease; *TN* is being identified as correctly without the disease; *FN* means detection incorrectly when the disease is present, and the model does not detect; *FP* means the disease is not present, but the model detects disease.

(5) $$ACC=\frac{TP+TN}{TP+FP+TN+FN}\times 100\%$$

(6) $$SEN=\frac{TP}{TP+FN}\times 100\%$$

(7) $$PPV=\frac{TP}{TP+FP}\times 100\%$$

(8) $$SPE=\frac{TN}{TN+FP}\times 100\%$$

(9) $$OA=\frac{\mathrm{Correctly}\;\mathrm{classified}\;\mathrm{instances}}{\mathrm{All}\;\mathrm{instances}}\times 100\%$$

## 4. Results

In this section, we first design several experiments to test the impact of each part of the proposed model and find the optimal parameters using a grid search algorithm (with respect to the overall accuracy). Then, many experiments are conducted under intra-patient and inter-patient conditions to verify the advantages of the proposed model. In addition, we compare the proposed model with state-of-the-art methods. Note that all the experiments were implemented in MATLAB R2018a and PyCharm 2018 run in Windows 7 on an Intel Core i7 CPU (@ 2.60 GHz) with a 1080 Ti GPU and 8 GB RAM.

### 4.1. Experiment Setup and Optimality Evaluation

In this subsection, some important hyperparameters are tested for the original DenseNet and multilevel DWT models. This approach can provide guidelines for the determining of the optimal parameters of the final model. First, we designed several experiments for the inter-patient paradigm and used 1/4 of the data set to verify the effects of input segments and the optimal structure of MDD-Net. Then, we chose the preferable parameters as a list through an analysis of the test results. Finally, we obtained the best parameters using a grid search algorithm.

#### 4.1.1. Impact of Input Segments

We use an adaptive frequency detection method (ASFS) to generate the inputs of the network. As mentioned above, a single segment contains multiple periodic rhythms. We need to find the length of the segment that has the best performance. Notably, the longer a segment is, the greater the amount of heartbeat discrimination information that can be provided; additionally, if a segment is too long, the network input may be too large to process. Hence, we confined the length of a segment to 10,000 sample points with an interval of 1000 and tested the performance of the original DenseNet model [34]. Note that other hyperparameters remain consistent, as shown in Table 7.

**Figure 6:**
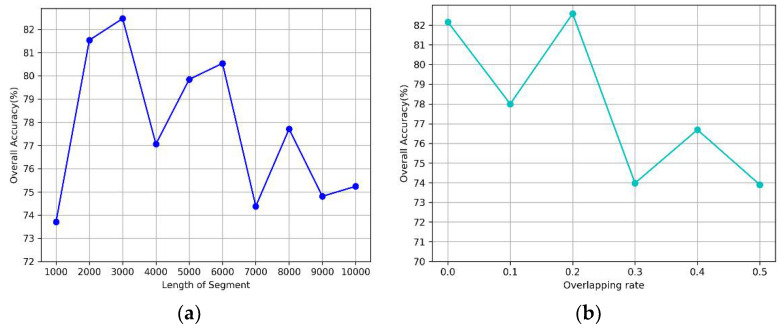
The impact of input segment on performance. (**a**) the length, (**b**) the overlapping rate.

From Figure 6a, we can observe that the model achieves good performance when the length of a segment is 2000 or 3000. Moreover, the accuracy significantly decreases when the length is over 6000. Additionally, we evaluated the effect of the segment overlap rate using a segment length of 2000. As shown in Figure 6b, the accuracy changed rapidly with an increasing overlap rate and decreased dramatically when the overlap exceeded 0.2. The model yields good results when the value is between 0.1 and 0.3.

#### 4.1.2. The Optimality Evaluation on the Structure of the MDD-Net

**Figure 7:**
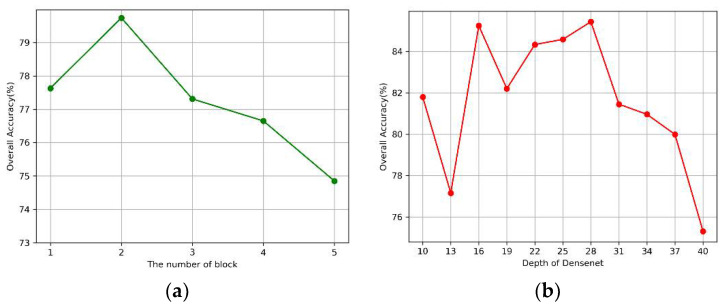
The impact of the original DenseNet. (**a**) Blocks, (**b**) Depth of the network.

**Figure 8:**
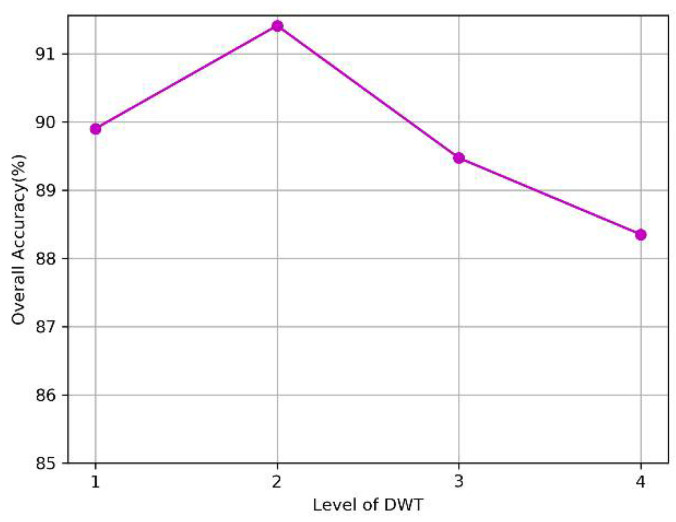
The impact of Multilevel-DWT.

Our proposed model consists of the DenseNet model and the multilevel DWT model. First, we investigated the effects of the number of dense blocks and network depth on the performance of the DenseNet model. As for the multilevel DWT model, we independently tested the impact of each DWT level. The results are presented in Figure 7 and Figure 8 respectively. Note that we used the parameters listed in Table 7 when we tested the effect of one single variable.

As shown in Figure 7a, the model can extract a sufficient number of features for classification when the number of blocks is less than 3, but the accuracy decreases rapidly when the value exceeds 3, which may be due to the overfitting of the training model. In Figure 7b, the model achieved relatively good results when the depth was between 16 and 28. In Figure 8, an improvement was observed in classification performance at the second level of decomposition compared to that at the first level of decomposition. This finding suggests that the combined multilevel DWT method can better extract the features of signals. However, we did not observe an improvement when we increased the decomposition from the 3rd to the 4th level.

In summary, we obtained the optimal parameters after the initial screening. Next, we will determine the optimal combination of parameters using the grid search algorithm. Note that we tested the proposed model and used 2000 instances as the validation set. The optimal parameters are shown in Table 8. Notably, single-model optimal parameters are not necessarily global optimal parameters due to the differences in features. We finally obtained 3454 normal, 15,011 MI, 11,339 CAD, and 22,215 CHF segments after determining the best parameters of the ASFS. Using these optimum parameters from Table 8, the next set of experimental investigations was performed.

### 4.2. Results of Automated Detection Based on Intra-Patient Paradigm 

**Figure 9:**
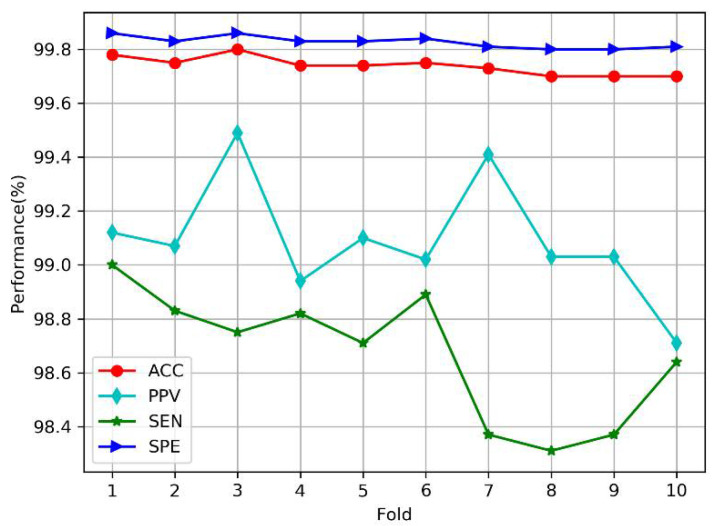
Plots of performance measures versus the number of folds under intra-patient paradigm.

Under the intra-patient paradigm, we performed 10-fold cross-validation according to the method of Acharya et al. [23]. All segments (3454 normal, 15,011 MI, 11,339 CAD, and 22,215 CHF) were divided into 10 parts almost equally. For each step, 9/10 segments were selected for training, and the rest were used for testing. Figure 9 shows the plots of the average performance measures based on the number of steps (or folds) in MDD-Net. The accuracy and specificity of all folds are high (above 99.70%), which indicates that the proposed model can accurately perform cardiovascular detection under the intra-patient paradigm. Furthermore, the variations in the four indicators, including the accuracy (99.70~99.78%), sensitivity (98.31~99.00%), and specificity (99.80~99.86%), are less than 1%, which indicates that our model is stable.

In Table 9, we present the overall confusion matrix for cardiovascular detection based on 10-fold cross-validation. The average accuracy, positive predictive value, sensitivity, and specificity were 99.74%, 99.09%, 98.67%, and 99.83%, respectively. The results show that for the CAD group, only a few samples (0.1%) were misclassified as CHF. In the MI group, 0.01% of the cases were misclassified as CAD, and in the CHF group, 0.03% of the cases were incorrectly classified as CAD, reflecting high classification performance.

### 4.3. Results of Automated Detection Based on Inter-Patient Paradigm

For the inter-patient paradigm, the classification performance of each method was evaluated based on the training instances from DS1 (Table 2), and the method was then tested with the instances from DS2. In Table 10, we show the performance of different models, including DenseNet, multilevel DWT, and MDD-Net. Note that the hyperparameters used are the same as those shown in Table 8. The results suggest that the proposed model performs better in classification than do other models. Notably, the proposed model displayed competitive performance (average accuracy of 96.92%, positive predictive value of 92.17%, sensitivity of 89.18%, and specificity of 97.77%).

**Figure 10:**
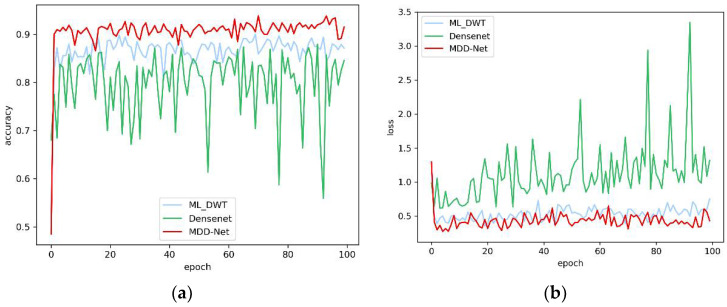
The overall accuracy and loss curves of models on the test set. (**a**) Overall accuracy; (**b**) Loss.

Furthermore, we compared the accuracy and loss of the three models based on the test set during the training process, as shown in Figure 10. We can easily observe that an oscillation phenomenon occurs during the training process for the DenseNet model. The ML-DWT model displays better stability than DenseNet, but the overall accuracy of the model was below the target range. Only the proposed model, which combines the other two models, exhibits sufficient stability and accuracy. In addition, the proposed model achieves a faster convergence speed than the other models. Therefore, the combination of features improves precision and stability.

### 4.4. Results of Robustness to Noise

**Figure 11:**
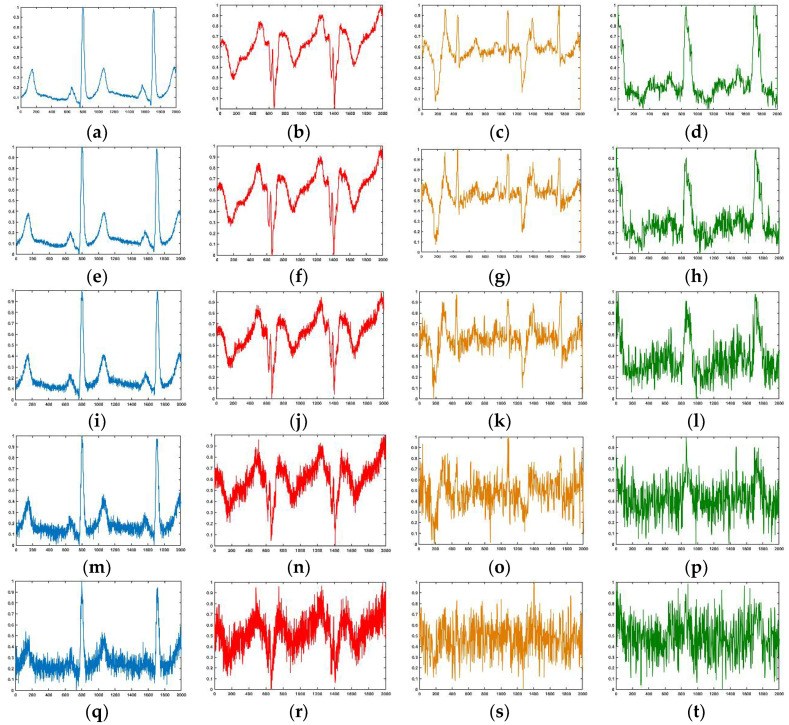
The noisy segment with different SNR. From left to right: Normal, MI, CAD, and CHF. (**a**–**d**) SNR = 24 dB; (**e**–**h**) SNR = 18 dB; (**i**–**l**) SNR = 12 dB; (**m**–**p**) SNR = 6 dB; (**q**–**t**) SNR = 0 dB.

In a real-life production environment, the ECG signal often contains different levels of noise. Hence, we tested the performance of our model under different levels of noise. Note that we used the awgn function in the MATLAB toolbox to generate different levels of white noise and employed the signal-to-noise ratio (SNR) to evaluate the level of noise. Figure 11 shows the waveforms of the normal, MI, CAD, and CHF segments with different levels of Gaussian white noise. The figure shows that when the SNR of the signal is less than 12 dB, the morphological characteristics of the waveform are generally ambiguous; especially the CAD and CHF waveforms are seriously damaged. When the SNR is 0 dB, the waveforms of all diseases are highly disrupted and difficult to distinguish with the naked eye.

**Figure 12:**
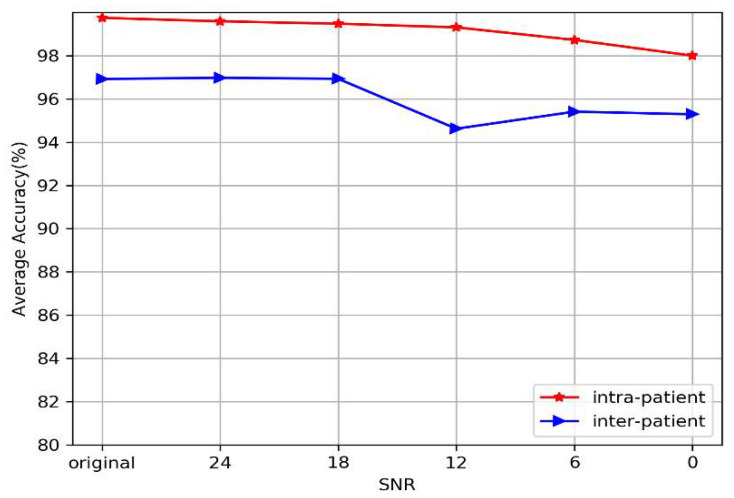
Plots of average performance of our model at different SNRs under intra-patient and inter-patient paradigms.

Table 11 and Figure 12 show the average performance of the proposed model at different SNRs under intra-patient and inter-patient conditions. Table 11 indicates that the performance of the model slightly decreases as the strength of noise increases, but it still maintains high performance under both the intra-patient paradigm and the inter-patient paradigm. The classification accuracy exceeds 99.31% when the SNR is greater than 12 dB under the intra-patient paradigm. Specifically, our model still achieved an accuracy of 98%, even though the SNR of the signal is 0 dB. For the inter-patient paradigm, the classification accuracy of our model is almost the same as that of the original signal when the SNR exceeds 12 dB (96.93~96.98%), except that the PPV and SEN decrease slightly. When the SNR is 0 dB, our model still achieves an accuracy of 95%. In summary, we can directly see from Figure 12 that our model performs stably under different levels of noise, whether under the intra-patient or inter-patient paradigm. The experiments show that the proposed model can achieve fairly good performance for different kinds of noise and various SNRs.

### 4.5. Results of Robustness to Imbalance Category 

In reality, the proportion of patients with diseases is often much smaller than the proportion of healthy patients. To effectively simulate and explain reality, we keep the number of normal cases unchanged and decrease the number of patients with diseases proportionally, as shown in Table 12. 

For the disease instances in the test set, such as CAD, only 11 were considered per fold (3% of normal) under the intra-patient paradigm when the scale was 100. In this paper, we adopted the Borderline-SMOTE algorithm to generate representative minority samples and added them to the training set. In addition, we used the *FL* function to solve the category imbalance problem by reducing the internal weights of simple samples. Note that the number of test sets remains constant during each experiment.

In the case of unbalanced categories, we focus on the performance of disease classes. Table 13 shows the confusion matrix and classification performance of diseases under the intra-patient and inter-patient paradigms. The sensitivity and precision of MI decreased to some extent with increasing scale under the intra-patient paradigm. However, the other indicators remained high. In particular, most of the performance indexes reach nearly 99% for CAD and CHF classification. For instance, in the CHF group, the proposed system yielded 99.67% accuracy, a 95.63% positive predictive value, 98.65% sensitivity, and 99.73% specificity when the scale was set to 100. Under the inter-patient paradigm, as the scale increased, the accuracy of disease classification increased. However, we can see that the performance for MI is more easily affected by the scale than is the performance for CAD or CHF, and the sensitivity of our model was not ideal for MI when the scale exceeded 20. However, the proposed model can effectively detect CHF and CAD. Acceptable performance (accuracy of 98.83%, positive predictive value of 92.80%, sensitivity of 89.92%, and specificity of 99.49%) for CHF was achieved even when the scale was set to 80.

**Figure 13:**
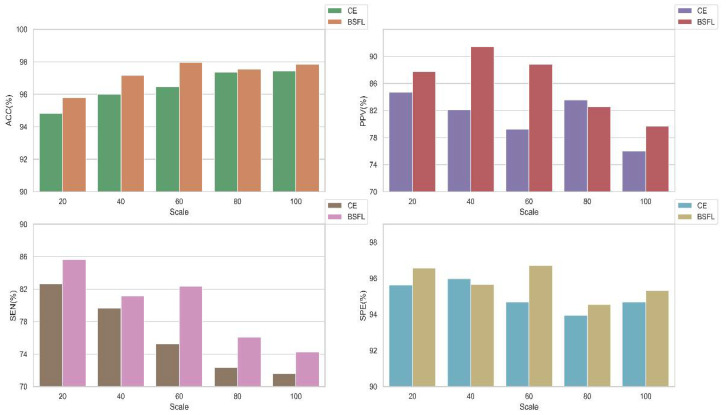
The average performance bar of the model with and without the algorithms in different scales under inter-patient paradigm. BSFL: Borderline-SMOTE algorithm and focal loss function. CE: cross-entropy loss function.

To prove the validity of our hybrid methods described, we conducted experiments with and without the algorithms in this work. The average performances of the models at different scales are shown in Figure 13. It can be observed that the performance of the model using the algorithm is better than those without the algorithm. In particular, the results of sensitivity and positive predictive value using the algorithm are obviously better than the other. In summary, our method can better detect diseases from imbalanced data sets.

### 4.6. Comparison of Other Deep Learning Models

In this paper, we used the same input segments and evaluated several popular deep learning models, as shown in Table 14. Note that we used the network structure as described in the original paper. DenseNet used the same network and parameters as the proposed model, as shown in Table 8. We can see that almost all models achieved good classification performance under the intra-patient paradigm, of which VGG_16 obtained the best result (accuracy of 99.84, positive predictive value of 99.62, sensitivity of 99.44, and specificity of 99.89%). The proposed input generation algorithm can provide distinguishable features. However, the classification performance varies greatly under inter-patient paradigms. For the inter-patient paradigm, VGG_16 achieved the worst classification performance. The proposed model performed well for both paradigms and achieved the best classification performance under inter-patient conditions. In addition, the performance of DenseNet was better than that of VGG_16 or ResNet, which is the reason we selected DenseNet as the basis of the proposed model.

## 5. Discussion

The purpose of this study is to propose a novel single-lead cardiovascular disease classification method that requires simple preprocessing effort and still has the same level of performance as or better performance than other popular methods. Notably, we hope to make a breakthrough for the inter-patient paradigm. Here, we summarize the key features of our model and discuss the advantages and disadvantages of the proposed model compared with the related literatures shown in Table 15.

First, we propose a simple ASFS approach to generate inputs without employing conventional data preprocessing steps, such as domain-specific feature extraction for noise removal or the R-peak location algorithm. Unlike our method, many existing methods [11,12,22,23] rely on various preprocessing steps to achieve high classification performance. Note that although they require data preprocessing steps, none of the methods yields better results than our method except for sensitivity under the intra-patient paradigm, as shown in Table 15. For the inter-patient paradigm, the proposed model could almost achieve the best classification results than those of most of the investigated literatures. Our model also achieved acceptable performance (accuracy of 96.92%, positive predictive value of 92.17%, sensitivity of 89.18%, and specificity of 97.77%) for 4-class cardiovascular disease classification (normal, CAD, MI, and CHF). To our knowledge, this is the first work reporting 4-class classification under the inter-patient paradigm. 

Second, to further test the classification performance of the proposed model in a multilevel noise environment, we added multilevel Gaussian noise to the original signals. The impact of multilevel noise is illustrated in Table 11 and Figure 12, in which the performance of the classification model changes little at different noise levels under both the intra-patient paradigm and inter-patient paradigm. Normally, the useful ECG signal appears as a low-frequency part of the signal or a relatively stable signal, and the noise signal appears as a high-frequency signal. The high-frequency Gaussian noise can be filtered out of a signal when multilevel 2D-DWT is performed. In addition, the multilayer convolutional structure improves the ability of the model to filter noise and mine useful information from ECGs. Hence, the proposed model exhibits good robustness to noise.

Third, we use the original input ECG data with an imbalance between normal and disease categories. To overcome the problem of category imbalance, on the basis of studies [39,40], we used a hybrid method to increase the training set and changed the sample batch weights to optimize our model. We adopted the Borderline-SMOTE algorithm to add minority samples to the training set; additionally, the *FL* function was employed to solve the category imbalance problem by reducing the internal weights of simple samples. In Table 13, our model yielded remarkable performance at different imbalanced scales under the two paradigms. Under the intra-patient paradigm, our method achieved the highest accuracy at 98.88% for MI, 99.70% for CAD, and 99.67% for CHF, even though the scale was 100. This finding reflected acceptable model performance under the inter-patient paradigm. In Figure 13, we demonstrate the validity of our method by comparing the performance of two classification models obtained using the same inputs with and without the Borderline-SMOTE algorithm and *FL* function.

Finally, we explained why DenseNet was chosen as the core part of our model by comparing several popular deep learning network frameworks. In Table 14, the performance difference among several popular learning frameworks under the intra-patient paradigm is shown to be minimal, but the performance under the inter-patient paradigm varies greatly, and the DenseNet model performed better than other deep learning models (accuracy of 93.97%, positive predictive value of 87.78%, sensitivity of 81.77%, and specificity of 95.24%).

The main highlights of our proposed algorithm are as follows:

(1) A novel ASFS algorithm is proposed. The algorithm can generate effective inputs without conventional data preprocessing (noise removal and R-peak location).
(2) Compared with traditional deep learning algorithms, our combined model has small steady-state error and achieved superior results.
(3) Our model has good robustness to noise and can overcome category imbalance.
(4) The proposed work has considerable practical significance considering the performance of the proposed model under the inter-patient paradigm.

However, we should also mention that during the training phase, our method requires a large number of heartbeat data sets that must be annotated by clinical experts. In the medical field, it is difficult to obtain such data sets with abnormal patterns. In addition, the sensitivity for MI under the inter-patient paradigm needs to be improved.

## 6. Conclusions

In this paper, we presented a novel and effective model (MDD-Net) for the detection of cardiovascular disease. The ASFS algorithm is employed to obtain consistent input segments without using regular preprocessing operations. We concatenate abstract and time-frequency features to obtain the resultant combined feature vector. Our model achieved higher stability and accuracy than the solo-feature DenseNet model. According to the results of the experiments, the proposed model significantly outperforms the existing algorithms in the literature for both intra-patient and inter-patient paradigms. Specifically, the model achieved an average accuracy of 96.92%, positive predictive value of 92.17%, sensitivity of 89.18%, and specificity of 97.77% under the inter-patient paradigm, which is of practical significance. Moreover, our model has good robustness to noise and imbalanced classes. Therefore, the proposed approach will be a useful component of clinical decision support systems for cardiologists.

In future work, we will improve the performance of our model and expand the predicted disease types under the inter-patient paradigm using more ECG data. Specifically, the performance of MI needs to be improved. Using more ECG data means more disease type labels. However, annotating disease types is very expensive and time-consuming. We want to develop a semi-supervised heartbeat classification model by using a large amount of unannotated ECG databases. Hence, we will work on developing an activated learning classification system to solve this problem. The ultimate goal of our work is to design a cloud version of the proposed method and apply it by using mobile devices to provide reliable and practical diagnostic results.

## Figures and Tables

**Table 1:** Literature reviews using ECG for detection of normal, MI, CAD, and CHF.

Author (Year)	Database	Feature Extraction Method (Classifiers)	Intra-Patient	Inter-Patient
Normal and CAD				
Acharya et al., (2017) [8]	Fantasia and St.Petersburg databases	HOS (KNN/DT)	ACC = 98.99%	
			SEN = 97.75%	
			SPE = 99.39%.	
Kumar et al., (2017) [9]	Fantasia and St.Petersburg databases	FAWT (LS-SVM)	ACC = 99.60%	
			SEN = 99.57%	
			SPE = 99.61%	
Normal and MI				
Baloglu et al., (2019) [10]	PTB diagnostic ECG database	CNN (Softmax)	ACC = 99.78%	
Han et al., (2019) [11]	PTB diagnostic ECG database	Energy entropy based on MODWPT; Feature fusion (SVM)	ACC = 99.75%	ACC = 92.69%
			SEN = 99.37%	SEN = 80.96%
			PPV = 99.70%	PPV = 86.14%
Sharma et al., (2018) [12]	PTB diagnostic ECG database	Wavelet decomposition based on biorthogonal filter bank, fuzzy entropy (KNN)	ACC = 99.62%	
			SEN = 99.76%	
			SPE = 99.12%	
Acharya et al., (2017) [13]	PTB diagnostic ECG database	11-layer CNN (Softmax)	ACC = 95.22%	
			SEN = 95.49%	
			SPE = 94.19%	
Reasat et al., (2017) [14]	PTB diagnostic ECG database	CNN with inception block (Softmax)		ACC = 84.54%
				SEN = 85.33%
				SPE = 84.09%
Sharma et al., (2017) [15]	PTB diagnostic ECG database	SWT Sample entropy, log energy entropy, and median slope; (SVM/KNN)	ACC = 98.84%	ACC = 81.71%
			SEN = 99.35%	SEN = 79.01%
			SPE = 98.29%	SPE = 79.26%
Padhy et al., (2017) [16]	PTB diagnostic ECG database	SVD (SVM)	ACC = 95.30%	
			SEN = 94.60%	
			SPE = 96.00%	
Acharya et al., (2016) [17]	PTB diagnostic ECG database	DWT (KNN)	ACC = 98.8%	
			SEN = 99.45%	
			SPE = 96.27%	
Normal and CHF				
Acharya et al., (2019) [18]	MITBIH Normal Sinus Rhythm, BIDMC CHF database	1D-CNN (Softmax)	ACC = 98.97%	
			SEN = 98.87%	
			SPE = 99.01%	
Sudarshan et al., (2017) [19]	MIT-BIH Normal Sinus Rhythm Database, BIDMC CHF database	Dual tree complex wavelet transform (KNN)	ACC = 99.86%	
			SEN = 99.78%	
			SPE = 99.94%	
Subasi et al., (2013) [20]	BIDMC CHF database, MIT-BIH Arrhythmia database	Autoregressive (AR) Burg (C4.5 DT)	SEN = 99.77%	
			SPE = 99.93%	
Normal, CAD and MI				
Acharya et al., (2017) [21]	St.Petersburgdatabases, PTB diagnostic ECG database,	DWT EMD DCT (KNN)	ACC = 98.5%	
			SEN = 98.5%	
			SPE = 99.7%	
Normal, CAD, MI, and CHF				
Fujita et al., (2017) [22]	St.Petersburg databases, PTB diagnostic ECG database, BIDMC CHF database	WPD ReliefF (KNN)	ACC =97.98%	
			SEN = 99.61%	
			SPE = 94.84%	
Acharya et al., (2017) [23]	St.Petersburg databases, PTB diagnostic ECG database, BIDMC CHF database	CWT Contourlet Transform Shearlet Transform (DT KNN)	ACC = 99.55%	
			SEN = 99.93%	
			SPE = 99.24%	

ACC: Accuracy, SEN: Sensitivity, SPE: Specificity, HOS: Higher-Order Statistics and Spectra, PCA: Principle Component Analysis, SVD: Singular Value Decomposition, LS-SVM: Least Squares Support Vector Machine, DWT: Discrete Wavelet Transform, FAWT: Flexible Analytic Wavelet Transform, SWT: Stationary Wavelet Transform, DCT: Discrete Cosine Transform, CWT: Continuous Wavelet Transform, EMD: Empirical Mode Decomposition, DT: Decision Tree, KNN: K-Nearest Neighbors, CNN: Convolution Neural Network.

**Table 2:** Summary of data used in this paper.

Database	Diagnosis Type	Used Lead°	Sampling Rate (Hz)	Subjects	Records
St-Petersburg	CAD	II	257	7	17
BIDMC CHF	CHF	II	250	15	15
PTB Diagnostic	Normal	II	1000	52	80
	MI	II	1000	148	368

**Table 3:** The details of the data distribution scheme.

Paradigm	Class	Training Set (DS1)	Testing Set (DS2)
Inter-patient	Normal	104, 105, 116, 117, 121, 122, 131, 150, 155, 156, 165, 166, 169, 170, 172, 173, 174, 180, 182, 184, 185, 198, 214, 229, 233, 234 (26 persons)	235~248, 251, 252, 255, 260, 263, 264, 266, 267, 276, 277, 279, 284 (26 persons)
	MI	001~074 (74 persons)	75~103, 108, 111, 120, 128, 135, 138~142, 145, 148, 149, 152, 158, 160, 163, 183, 189, 193, 195, 197, 205, 207, 211, 223, 226, 230, 231, 259, 261, 265, 268, 270, 273, 274, 280, 282, 283, 287, 290~294 (74 persons)
	CAD	001, 010, 016, 017 (4 persons)	020, 025, 031 (3 persons)
	CHF	001~008 (8 persons)	009~015 (7 persons)
Intra-patient	All data were chosen randomly as training and test samples. 10-fold cross-validation was employed, 9/10 of data was selected for training and the remaining data was used for testing.		

**Table 4:** The implementation flow of the ASFS.

Input:	The raw ECG data $V_{e}$, current sample frequency $F_{cur}$, Max sample frequency $F_{\max}$, the number of ECG cycles in one segment $N_{cyc}$, overlapping rate of the segment $R_{s}$
Output:	The matrix of segments $M_{s}$
Step 1:	Calculate the length of a desirable segment $L_{s}=F_{cur}\times N_{cyc}$
Step 2:	Calculate the length of the overlap $Lo=ceil\left(L_{s}\times R_{s}\right)$
Step 3:	Calculate the length of input ECG $L_{e}=size\left(V_{e},2\right)$
Step 4:	For the loop of segment extraction from the raw ECG
Step 5:	Intercept from the raw ECG $V_{e}$ and get the segment $seg=V_{e}\left(1:L_{s}\right)$
Step 6:	Get the expected segment based on the current frequency $F_{cur}$ and expected frequency $F_{\max}$;$seg=resample\left(seg,F_{\max},F_{cur}\right)$
Step 7:	Normalize the segment
Step 8:	Add the normalized segment to the matrix $M_{s}$;
Step 9:	Calculate the new $V_{e}$ based on the length of input ECG $L_{e}$ and the length of overlap $L_{o}$ after intercepting the segment $V_{e}=V_{e}\left(\left(L_{s}-L_{o}+1\right):end\right)$
Step 10:	End for
Step 11:	Get the desirable matrix of segments $M_{s}$

**Table 5:** The network architecture of our MDD-Net model.

Layer	Output Shape	Filter (Kernel size, Stride Size, Number)
Convolution2D	(None,60,50,24)	3×3,1×1,24
AveragePooling2D	(None,30,25,24)	3×3,2×2
Dense block 1	(None,30,25,36)	$\left[\begin{array}{ll} 3\times 3,1\times 1,48 & \mathrm{conv}\\ 3\times 3,1\times 1,12 & \mathrm{conv} \end{array}\right]\times 3$
Transition block 1	(None,15,13,18)	$\left[\begin{array}{cc} 3\times 3,1\times 1,30 & \mathrm{conv}\\ 3\times 3,2\times 2 & \mathrm{pooling} \end{array}\right]$
Dense block 2	(None,15,13,30)	$\left[\begin{array}{ll} 3\times 3,2\times 2,48 & \mathrm{conv}\\ 3\times 3,2\times 2,12 & \mathrm{conv} \end{array}\right]\times 3$
Transition block 2	(None,8,7,15)	$\left[\begin{array}{cc} 3\times 3,1\times 1,33 & \mathrm{conv}\\ 3\times 3,2\times 2 & \mathrm{pooling} \end{array}\right]$
Dense block 3	(None,8,7,27)	$\left[\begin{array}{ll} 3\times 3,2\times 2,48 & \mathrm{conv}\\ 3\times 3,2\times 2,12 & \mathrm{conv} \end{array}\right]\times 3$
Maxpooling2D	(None,4,4,27)	3×3,2×2
Conv block 1	(None,30,25,24)	$\left[\begin{array}{ll} 3\times 3,1\times 1,96 & \mathrm{conv}\\ 3\times 3,1\times 1,24 & \mathrm{conv} \end{array}\right]$
Concatenation 1	(None,30,25,96)	None
Maxpooling2D	(None,15,13,96)	3×3,2×2
Conv block 2	(None,15,13,12)	[3×3,1×1,12 conv]
Conv block 3	(None,15,13,24)	$\left[\begin{array}{ll} 3\times 3,1\times 1,96 & \mathrm{conv}\\ 3\times 3,1\times 1,24 & \mathrm{conv} \end{array}\right]$
Concatenation 2	(None,15,13,108)	None
Maxpooling2D	(None,8,7,108)	3×3,2×2
Conv block 4	(None,8,7,12)	[3×3,1×1,12 conv]
Conv block 5	(None,8,7,24)	$\left[\begin{array}{ll} 3\times 3,1\times 1,96 & \mathrm{conv}\\ 3\times 3,1\times 1,24 & \mathrm{conv} \end{array}\right]$
Concatenation 3	(None,8,7,108)	None
Maxpooling2D	(None,4,4,108)	3×3,2×2
Concatenation 4	(None,4,4,135)	None
Maxpooling2D	(None,2,2,135)	3×3,2×2
GlobalMaxPooling2D	(None,135)	None
Softmax	4	None

**Table 6:** The implementation flow of the Borderline-SMOTE.

Input:	The original training set F, the majority-class set $S_{\min}=\left\{f_{1},f_{2},\ldots ,f_{n}\right\}$
Output:	The new training set $F_{o}$ after using Borderline-SMOTE algorithm
Step 1:	Calculate the k nearest neighbors of each sample in the minority set $S_{\min}$
Step 2:	Classify the samples in $S_{\min}$ according to these k nearest neighbors (a) if the k nearest neighbors of a sample are all majority-class samples, we define this sample as a noise sample and place it in the $N^{\prime }$ set. (b) if the k nearest neighbors of a sample are all minority-class samples, we define this sample as a safe sample and place it in the S set. (c) if the k nearest neighbors of a sample have both majority-class samples and minority-class samples, this sample is considered a boundary sample and is put into the B set.
Step 3:	For loop until the number of artificial minority-class samples is met.
Step 4:	Set the boundary sample set $B=\left\{f_{1}{}^{\prime },f_{2}{}^{\prime },\ldots ,f_{n}{}^{\prime }\right\}$, calculate k nearest neighbors in the minority-class set $S_{\min}$ of each sample $f_{i}{}^{\prime },i=1,2,\ldots ,n$ in the B set, and compose the set $f_{ij}$.
Step 5:	Randomly select s(1<s<n) nearest neighbors.
Step 6:	Calculate the difference of all attributes between a sample and its nearest neighbors $d_{ij}=f_{i}{}^{\prime }-f_{ij},\;j=1,2,\ldots ,s$.
Step 7:	The attribute difference multiplied by a random number $r_{ij},r_{ij}\in \left(0,1\right)$. If $f_{ij}$ is a sample in the $N^{\prime }$ or S set, then $r_{ij}\in \left(0,0.5\right)$.
Step 8:	The generated artificial minority-class sample is $h_{ij}=f_{i}{}^{\prime }+r_{ij}*d_{ij},\;j=1,2,\ldots ,s$.
Step 9:	Add the generated sample to the new training set $F_{o}$
Step 10:	End for
Step 11:	Get the desirable training set $F_{o}$

**Table 7:** The consistent parameters of the original DenseNet.

Parameter	Value
The number of dense blocks	3
The depth of the network	13
Batch size	50
Epoch	50
Growth rate	12

**Table 8:** Grid parameter list and the optimal parameters of MDD-Net.

Item	Parameter	Alternative List	Best
The input segment	Segment length	(2000, 3000)	3000
	Overlapping rate	(0.1, 0.2, 0.3)	0.1
Reformed DenseNet	Batch size	(20, 30, 40, …, 200)	50
	Epoch	(100, 150, 200)	100
	Dense blocks	(1, 2, 3)	3
	Depth	(10, 13, 16, 19, …, 46)	10
	Growth rate	(12, 24)	12
Multilevel DWT	The level of DWT	(1, 2, 3)	3

**Table 9:** The overall classification results for cardiovascular detection across 10-fold.

		Predicted				ACC (%)	PPV (%)	SEN (%)	SPE (%)
		Normal	MI	CAD	CHF				
Original	Normal	**3297**	157	0	0	99.54	97.57	95.45	99.83
	MI	82	**14921**	2	6	99.52	98.95	99.40	99.57
	CAD	0	1	**11322**	16	99.95	99.92	99.85	99.98
	CHF	0	0	7	**22208**	99.94	99.90	99.97	99.93
	Average					**99.74**	**99.09**	**98.67**	**99.83**

**Table 10:** The results for cardiovascular detection under inter-patient paradigm.

Original/Predicted		Predicted				ACC (%)	PPV (%)	SEN (%)	SPE (%)
		Normal	MI	CAD	CHF				
DenseNet	Normal	**1075**	537	6	0	96.67	83.08	66.44	98.97
	MI	202	**5744**	20	290	94.00	86.94	91.82	94.82
	CAD	9	69	**3421**	1170	93.59	93.96	73.27	98.79
	CHF	8	257	194	**9908**	91.62	87.16	95.57	88.36
	Average					93.97	87.78	81.77	95.24
Multilevel DWT (ML-DWT)	Normal	**1322**	295	1	0	96.69	74.10	81.71	97.83
	MI	361	**5829**	16	50	96.81	95.04	93.17	98.17
	CAD	91	7	**3717**	854	93.10	85.55	79.61	96.56
	CHF	10	2	611	**9744**	93.33	91.51	93.99	92.79
	Average					94.98	86.55	87.12	96.34
**MDD-Net (proposed)**	Normal	**1173**	442	3	0	97.32	87.41	72.50	99.21
	MI	149	**6013**	48	46	96.95	92.97	96.12	97.27
	CAD	15	10	**4250**	394	96.67	92.49	91.03	98.11
	CHF	5	3	294	**10065**	96.76	95.81	97.09	96.49
	Average					**96.92**	**92.17**	**89.18**	**97.77**

**Table 11:** The average performance of different SNRs under intra-patient and inter-patient paradigms.

SNR/Paradigm	Intra-Patient				Inter-Patient			
	ACC (%)	PPV (%)	SEN (%)	SPE (%)	ACC (%)	PPV (%)	SEN (%)	SPE (%)
Original	99.74	99.09	98.67	99.83	96.92	92.17	89.18	97.77
24 db	99.59	97.97	98.51	99.75	96.98	90.74	89.59	98.05
18 db	99.48	97.65	97.91	99.67	96.93	88.71	89.20	98.08
12 db	99.31	97.95	96.59	99.53	94.62	83.12	84.19	96.61
6 db	98.73	95.48	94.02	99.13	95.41	84.06	86.96	97.16
0 db	98.00	93.16	90.87	98.62	95.29	82.37	82.85	97.02

**Table 12:** The number of instances of each category in different scales.

Scale	Normal	MI	CAD	CHF
Original	3454	15,011	11,339	22,215
20	3454	750	566	1110
40	3454	375	283	555
60	3454	250	188	370
80	3454	187	141	277
100	3454	150	113	222

**Table 13:** The confusion matrix and classification performance of diseases in different scales.

Scale	Intra-Patient							Inter-Patient						
	Confusion Matrix (10-Fold)				Performance (ACC PPV SEN SPE)			Confusion Matrix				Performance (ACC PPV SEN SPE)		
					MI	CAD	CHF					MI	CAD	CHF
20	**3415**	39	0	0	97.91	99.90	99.88	**1544**	74	0	0	94.18	97.20	97.39
	84	**666**	0	0	94.47	99.30	99.82	76	**230**	5	1	75.66	89.90	90.14
	0	0	**564**	2	88.80	99.65	99.55	1	0	**178**	54	73.72	76.39	97.10
	1	0	4	**1105**	99.24	99.92	99.96	0	0	15	**503**	96.88	99.18	97.46
40	**3429**	24	0	1	97.58	99.74	99.74	**1611**	7	0	0	95.86	98.37	98.51
	88	**286**	1	0	92.26	99.27	98.05	80	**74**	2	0	91.36	86.49	92.83
	0	0	**273**	10	76.27	96.47	99.82	1	0	**96**	19	47.44	82.76	94.98
	0	0	1	**554**	99.44	99.95	99.73	0	0	13	**246**	99.65	99.26	98.99
60	**3430**	24	0	0	98.15	99.86	99.86	**1605**	13	0	0	97.21	98.53	98.63
	55	**195**	0	0	89.04	98.40	99.19	38	**62**	3	1	82.67	86.36	88.77
	0	0	**185**	3	78.00	98.40	99.19	0	0	**57**	20	59.62	74.03	96.51
	0	0	3	**367**	99.40	99.93	99.92	0	0	6	**166**	99.30	99.52	98.83
80	**3440**	14	0	0	98.42	99.73	99.73	**1600**	18	0	0	96.55	98.57	98.83
	50	**137**	0	0	90.73	98.51	96.83	47	**31**	0	0	63.27	77.19	92.80
	0	0	**132**	9	73.26	93.62	99.28	5	0	**44**	9	39.74	75.86	89.92
	0	0	2	**275**	99.64	99.95	99.76	0	0	13	**116**	99.00	99.29	99.49
100	**3445**	9	0	0	98.88	99.70	99.67	**1599**	18	1	0	97.05	98.63	98.58
	35	**115**	0	0	92.74	98.10	95.63	34	**26**	0	2	59.09	78.38	83.48
	0	0	**103**	10	76.67	91.15	98.65	0	0	**29**	17	41.94	63.04	93.20
	1	0	2	**219**	99.76	99.95	99.73	0	0	7	**96**	98.98	99.55	98.90

**Table 14:** Comparison of different deep networks for classification of Normal, MI, CAD, and CHF.

Model	Intra-Patient				Inter-Patient			
	ACC (%)	PPV (%)	SEN (%)	SPE (%)	ACC (%)	PPV (%)	SEN (%)	SPE (%)
VGG_16 [30]	**99.84**	**99.62**	**99.44**	**99.89**	79.63	64.03	56.65	85.83
ResNet_18 [33]	99.79	99.44	99.24	99.85	91.27	80.21	75.72	93.26
ResNet_34 [33]	99.79	99.52	99.18	99.85	91.99	81.47	77.15	93.78
ResNet_50 [33]	99.72	99.25	99.12	99.80	89.76	75.63	76.08	92.58
DenseNet	99.63	99.20	98.86	99.73	93.97	87.78	81.77	95.24
**Proposed**	99.74	99.09	98.67	99.83	**96.92**	**92.17**	**89.18**	**97.77**

**Table 15:** Comparison of the proposed model against the recent literatures using the same databases.

Author (Year)	Intra-Patient				Inter-Patient			
	ACC (%)	PPV (%)	SEN (%)	SPE (%)	ACC (%)	PPV (%)	SEN (%)	SPE (%)
Normal and MI								
Han et al. (2019) [11]	99.75	99.70	99.37		92.69	86.14	80.96	
Sharma et al. (2018) [12]	99.62		99.76	99.12				
Sharma et al. (2017) [15]	98.84		99.35	98.29	81.71		79.01	79.26
Normal, CAD, MI, and CHF								
Fujita et al. (2017) [22]	97.98		99.61	94.84				
Acharya et al. (2017) [23]	99.55		**99.93**	99.24				
**Proposed**	**99.74**	**99.09**	98.67	**99.83**	**96.92**	**92.17**	**89.18**	**97.77**

## References

[1] Sayadi O., Shamsollahi M.B. (2007). Multiadaptive bionic wavelet transform: Application to ECG denoising and baseline wandering reduction. EURASIP J. Adv. Signal. Process..

[2] Buja L.M., Willerson J.T., Holmes David R.J. (2015). Coronary Artery Disease.

[3] Buja L.M., Willerson J.T. (1987). The role of coronary artery lesions in ischemic heart disease: Insights from recent clinicopathologic, coronary arteriographic, and experimental studies. Hum. Pathol..

[4] Gertsch M., Gertsch M. (2004). The Normal ECG and its (Normal) Variants BT–The ECG: A Two-Step Approach to Diagnosis.

[5] Chee J., Seow S.-C., Acharya U.R., Suri J.S., Spaan J.A.E., Krishnan S.M. (2007). The Electrocardiogram BT—Advances in Cardiac Signal Processing.

[6] Goldberger A.L., Gold-berger E. (1981). Clinical Electrocardiography, A Simplified Approach. Crit. Care Med..

[7] Madias J. (2006). ECG Changes in Response to Diuresis in an Ambulatory Patient with Congestive Heart Failure. Congest. Heart Fail..

[8] Acharya U.R., Sudarshan V.K., Koh J.E.W., Joy R., Hong J., Lih S., Muhammad A., Hagiwara Y., Rama M., Mookiah K. (2017). Biomedical Signal Processing and Control Application of higher-order spectra for the characterization of Coronary artery disease using electrocardiogram signals. Biomed. Signal. Process. Control..

[9] Kumar M., Bilas R., Acharya U.R. (2017). Biomedical Signal Processing and Control Characterization of coronary artery disease using flexible analytic wavelet transform applied on ECG signals. Biomed. Signal. Process. Control..

[10] Baran U., Talo M., Yildirim O., San R., Acharya U.R. (2019). Classification of myocardial infarction with multi-lead ECG signals and deep CNN. Pattern Recognit. Lett..

[11] Han C., Shi L. (2019). Automated interpretable detection of myocardial infarction fusing energy entropy and morphological features. Comput. Methods Programs Biomed..

[12] Sharma M., San R., Acharya U.R. (2018). A novel automated diagnostic system for classi fi cation of myocardial infarction ECG signals using an optimal biorthogonal fi lter bank. Comput. Biol. Med..

[13] Acharya U.R., Fujita H., Oh S.L., Hagiwara Y., Tan J.H., Adam M. (2017). Application of deep convolutional neural network for automated detection of myocardial infarction using ECG signals. Inf. Sci..

[14] Reasat T., Shahnaz C. Detection of inferior myocardial infarction using shallow convolutional neural networks. Proceedings of the 5th IEEE Reg. 10 Humanit. Technol. Conf. 2017, R10-HTC 2017.

[15] Sharma L.D., Sunkaria R.K. (2018). Inferior myocardial infarction detection using stationary wavelet transform and machine learning approach. Signal. Image Video Process..

[16] Padhy S., Dandapat S. (2017). Biomedical Signal Processing and Control Third-order tensor based analysis of multilead ECG for classification of myocardial infarction. Biomed. Signal. Process. Control.

[17] Acharya U.R., Fujita H., Sudarshan V.K., Oh S.L., Adam M., Koh J.E.W., Tan J.H., Ghista D.N., Martis R.J., Chua C.K. (2016). Automated detection and localization of myocardial infarction using electrocardiogram: A comparative study of different leads. Knowl. Based Syst..

[18] Acharya U.R., Fujita H., Lih S., Yuki O., Jen H., Tan H., Adam M., Tan R.S. (2019). Deep convolutional neural network for the automated diagnosis of congestive heart failure using ECG signals. Appl. Intell..

[19] Sudarshan V.K., Acharya U.R., Lih S., Adam M., Hong J., Kuang C., Poo K., San R. (2017). Automated diagnosis of congestive heart failure using dual tree complex wavelet transform and statistical features extracted from 2 s of ECG signals. Comput. Biol. Med..

[20] Mašeti Z., Subasi A. (2013). Detection of congestive heart failures using C4.5 Decision Tree. Southeast Eur. J. Soft Comput..

[21] Acharya U.R., Fujita H., Adam M., Lih O.S., Sudarshan V.K., Hong T.J., Koh J.E., Hagiwara Y., Chua C.K., Poo C.K. (2017). Automated characterization and classification of coronary artery disease and myocardial infarction by decomposition of ECG signals: A comparative study. Inf. Sci..

[22] Fujita H., Sudarshan V.K., Adam M., Oh S.L., Tan J.H., Hagiwara Y., Chua K.C., Chua K.P., Acharya U.R., Benferhat S., Tabia K., Ali M. (2017). Characterization of Cardiovascular Diseases Using Wavelet Packet Decomposition and Nonlinear Measures of Electrocardiogram Signal BT—Advances in Artificial Intelligence: From Theory to Practice.

[23] Acharya U.R., Fujita H., Sudarshan V.K., Oh S.L., Adam M., Tan J.H., Koo J.H., Jain A., Lim C.M., Chua K.C. (2017). Automated characterization of coronary artery disease, myocardial infarction, and congestive heart failure using contourlet and shearlet transforms of electrocardiogram signal. Knowl. Based Syst..

[24] de Chazal P., O’Dwyer M., Reilly R.B. (2004). Automatic classification of heartbeats using ECG morphology and heartbeat interval features. IEEE Trans. Biomed. Eng..

[25] Huang H., Liu J., Zhu Q., Wang R., Hu G. (2014). A new hierarchical method for inter-patient heartbeat classification using random projections and RR intervals. Biomed. Eng. Online.

[26] Luz E., Menotti D. How the choice of samples for building arrhythmia classifiers impact their performances. Proceedings of the 2011 Annual International Conference of the IEEE Engineering in Medicine and Biology Society.

[27] Goldberger A.L., Amaral L.A., Glass L., Hausdorff J.M., Ivanov P.C., Mark R.G., Mietus J.E., Moody G.B., Peng C.K., Stanley H.E. (2000). PhysioBank, PhysioToolkit, and PhysioNet: Components of a new research resource for complex physiologic signals. Circulation.

[28] Addison P.S. (2005). Wavelet transforms and the ECG: A review. Physiol. Meas..

[29] Daubechies I. (1990). The wavelet transform, time-frequency localization and signal analysis. Inf. Theory, IEEE Trans..

[30] Simonyan K., Zisserman A. Very Deep Convolutional Networks for Large-Scale Image Recognition. https://arxiv.org/pdf/1409.1556.pdf.

[31] Szegedy C., Liu W., Jia Y., Sermanet P., Reed S., Anguelov D., Erhan D., Vanhoucke V., Rabinovich A. Going Deeper with Convolutions. Proceedings of the IEEE Conference on Computer Vision and Pattern Recognition.

[32] Szegedy C., Vanhoucke V., Ioffe S., Shlens J., Wojna Z. Rethinking the Inception Architecture for Computer Vision. Proceedings of the IEEE Comput. Soc. Conf. Comput. Vis. Pattern Recognit.

[33] He K., Zhang X., Ren S., Sun J. Deep Residual Learning for Image Recognition. Proceedings of the IEEE Conference on Computer Vision and Pattern Recognition.

[34] Huang G., Liu Z., Van Der Maaten L., Weinberger K.Q. Densely connected convolutional networks. Proceedings of the 2017 IEEE Conference on Computer Vision and Pattern Recognition (CVPR).

[35] Chawla N.V., Bowyer K.W., Hall L.O., Kegelmeyer W.P. (2002). SMOTE: Synthetic Minority Over-sampling Technique. J. Artif. Intell. Res..

[36] Han H., Wang W.-Y., Mao B.-H., Huang D.-S., Zhang X.-P., Huang G.-B. (2005). Borderline-SMOTE: A New Over-Sampling Method in Imbalanced Data Sets Learning BT–Advances in Intelligent Computing.

[37] Lin T.Y., Goyal P., Girshick R., He K., Dollar P. (2020). Focal Loss for Dense Object Detection. IEEE Trans. Pattern Anal. Mach. Intell..

[38] Nasrabadi N.M., Bishop C.M. (1992). Pattern Recognition and Machine Learning.

[39] Acharya U.R., Oh S.L., Hagiwara Y., Tan J.H., Adam M., Gertych A., Tan R.S. (2017). A deep convolutional neural network model to classify heartbeats. Comput. Biol. Med..

[40] Sellami A., Hwang H. (2019). A robust deep convolutional neural network with batch-weighted loss for heartbeat classification. Expert Syst. Appl..

